# Multi-template imprinted solid-phase microextraction coupled with UPLC-Q-TOF-MS for simultaneous monitoring of ten hepatotoxic pyrrolizidine alkaloids in scented tea

**DOI:** 10.3389/fchem.2022.1048467

**Published:** 2022-11-28

**Authors:** Zhimin Luo, Xueqiang Chen, Yirong Ma, Fan Yang, Na He, Liangwei Yu, Aiguo Zeng

**Affiliations:** School of Pharmacy, Xi’an Jiaotong University, Xi’an, China

**Keywords:** hepatotoxic pyrrolizidine alkaloids, multi-template imprinting, solid phase microextraction, UPLC-Q-TOF-MS, scented tea

## Abstract

Pyrrolizidine alkaloids (PAs) are a series of ubiquitous natural toxins in flowering plants, which are associated with serious hepatic disease in humans. However, the simultaneously fast and sensitive monitoring of different PAs are still challenge because of the diversity of PAs and huge amount of interference in complex samples, such as scented tea samples. In this study, molecularly imprinted solid phase microextraction (MIP-SPME) fibers were fabricated by using multi-template imprinting technique for selective recognition and efficient enrichment of different PAs from scented teas. MIP-SPME could be used for selective adsorption of ten types of PAs through specific recognition cavity and strong ionic interaction, including senecionine, lycopsamine, retrorsine, heliotrine, lasiocarpine, monocrotaline, echimidine, erucifoline, europine and seneciphylline. The extraction parameters were also optimized including extraction time, elution solvent and elution time. Then, ultra performance liquid chromatography- quadrupole-time of flight mass spectrometry (UPLC-Q-TOF-MS) coupled with MIP-SPME method was developed for fast, simple, sensitive and accurate determination of ten PAs in scented teas. The established method was validated and presented satisfactory accuracy and high precision. It was also successfully applied for simultaneous determination of ten PAs in different scented tea samples. PAs were found in most of these scented tea samples, which suggest the cautious use of scented tea for consumers.

## Introduction

Tea is one of the most consumed flavored functional beverage in the world. Various types of tea are produced and consumed in many countries, such as green tea, black tea and scented tea (Ito et al., 2002). Scented tea is a series of reprocessed tea, which is a mixture of black, green, oolong, and other teas by mixing with various kinds of flowers, fruits, or plant spices to get different flavor (Lin et al., 2013). Because the aromatization of scented tea brings stronger pleasant odor, many consumers prefer to the scented tea. Therefore, the consumption of scented tea is increasing rapidly (Pokorný, 1995). However, a kind of natural toxins, pyrrolizidine alkaloids (PAs), could be stealthily brought in scented teas, which will highly threaten the human health.

PAs are secondary metabolites of plants and PAs have been identified in 3% of flowering plants and over 6,000 plants, but especially in *Asteraceae, Fabaceae, Orchidaceae, Apocynaceae* and *Boraginaceae* families (Mroczek et al., 2002). The first case of PA intoxication was because of the ingestion of PAs contaminated flour in 1920 (Willmot and Robertson, 1920). In 1988, Roulet reported the death of a newborn infant resulting from the consumption of PA-contaminated tea by the mother during pregnancy (Spang, 1989). Henceforth, the toxicity of PAs has been noted and many researches gradually verified their liver damage effect. The most important toxicological features of PAs are their hepatotoxicity and neurotoxicity. In addition, PAs can cause significant mutagenicity, carcinogenicity and embryotoxicity (Xia et al., 2018). Many intoxication cases have been reported and they were resulted from the consumption of PA-contaminated food or beverages (R, 1989; Roeder, 2000). Due to the toxic effect of PAs, the possible PAs-contaminated products have been monitored and PAs have been found in honey, milk, flour, salads, tea, herbal samples, etc., (Deinzer et al., 2002; Cheng et al., 2018; Chung and Lam, 2018). Specially, recent studies have reported that PAs were detected in different kinds of teas (Bodi et al., 2014; Mathon et al., 2014; Chung and Lam, 2018). On account of the addition of various flowers and plant spices, the scented tea would show much higher potential that was contaminated with PAs. Therefore, a high sensitive and accurate analysis method is required for sensitive determination of PAs in scented tea.

Due to the toxicity of PAs, the intake of PAs in foodstuffs have been strictly limited in many countries. A health based guidance value has been established and a recommended daily intake of PAs should be under 0.007 μg kg^−1^ body weight (Bodi et al., 2014). What is more, Austrian agency established more restrictive limits and demanded that any PAs containing products was forbidden (Mathon et al., 2014). Researchers have used many analytical techniques for the determination of trace amount of PAs in foodstuffs, such as HPLC (Wang et al., 2005), micellar electrokinetic chromatography (Yu et al., 2005), LC-MS (Mathon et al., 2014), GC-MS (Kowalczyk et al., 2017), etc., Before instrumental analysis, choosing a proper sample preparation method was the precondition and the key point for most of the analytical methods due to the complex matrix interference in plant samples (Yoon et al., 2015). Solid phase extraction (SPE) was the most frequently used sample preparation method for the separation and enrichment of targets in complex samples (Sierra and Morante-Zarcero, 2018). However, because of the limitation of time-consuming process, large sample requirement and significant use of organic solvents, SPE is gradually being replaced by solid phase microextraction (SPME) (Teixeira et al., 2020). On contrary, SPME is becoming more and more popular in sample preparation field because of the superior characteristics of miniaturized format, less sample requirement and solvent consumption, easy and fast usage (Pereira et al., 2022). Nevertheless, the major challenges SPME must face include the low capacity and poor selectivity because of the limited types of sorbent materials (Omarova et al., 2022).

In fact, the selection of sorbent material types greatly affect the extraction performance in both SPE and SPME. Therefore, researchers developed various materials for sorbent-based extraction, such as conductive polymers, molecularly imprinted polymers (MIPs), metal-organic frameworks and ionic liquids (Lashgari and Yamini, 2019; Li et al., 2019; Ma et al., 2022a; Ma et al., 2022b). Among these materials, MIPs are gained much attention because of their advantages of high stability, excellent selective adsorption property, resistance to a wide range of pH and solvents and ease of preparation (BelBruno, 2019). These capabilities were ascribed to the intrinsic properties of MIPs which are synthetic polymeric materials, and MIPs can provide complementary three dimensional cavities and binding sites to specifically recognize templates in complex matrix (Song et al., 2022). The use of MIPs in SPME technique has been widely reported for selective separation and enrichment of targets in environmental, food and biological analyses (Turiel and Martín-Esteban, 2019). MIPs synthesized in these studies often use a single template molecule to prepare and obtain polymers. Although these MIPs exhibit excellent specific selectivity for target analyte, the capacity was limited that simultaneously extract multicomponent targets (Mohiuddin et al., 2021). Therefore, researchers proposed a multi-template strategy, which two or more different templates were incorporated for the preparation of MIPs (Sreenivasan and Sivakumar, 1999). It has been proven that multi-template approaches not only can increase the capacity of MIPs for extraction of multiple targets but also can save time, energy and labor (Wang Y et al., 2020).

Practically, not all PAs are toxic and the toxicity of PAs was highly related with their structures. Only those PAs with un-saturated necine base and an allylic ester group are hepatotoxic, as exemplified by retrorsine, lasiocarpine, incidine, monocratoline, etc., (Xia et al., 2015), and these were summarized as hepatotoxic pyrrolizidine alkaloids (HPAs). Thereinto, the particular important hepatotoxic PAs include senecionine-type PAs, monocrotaline-type PAs, heliotrine-type PAs and lycopasmine-type PAs (EFSA, 2011). In term of the guideline of European Food Safety Authority (EFSA), we mainly focus on ten representative HPAs which have been identified as major hepatotoxins, including senecionine, lycopsamine, retrorsine, heliotrine, lasiocarpine, monocrotaline, echimidine, erucifoline, europine, and seneciphylline.

Considering the toxicity of HPAs, the sensitive and accurate method is required for the monitoring of HPAs in scented tea. In this work, MIP-SPME was prepared by using multi-template strategy, and then MIP-SPME coupled with UPLC-Q-TOF-MS method was established for sensitive and accurate determination of ten representative HPAs in popular scented tea. This work also provides information about PA-contaminated scented tea for the guidance of the cautious use of these PA-contaminated food.

## Experimental

### Reagents and apparatus

Analytical standards of lasiocarpine, heliotrine, monocrotaline, erucifoline, senecionine, europine, seneciphylline, echimidine and retrorsine (all with purity ≥ 95%) were purchased from ChemFaces (Wuhan, China). Analytical standards (all with purity ≥ 95%) of lycopsamine, luteolin, sinomenine, berberine hydrochloride and naringin were purchased from Toronto Research Chemicals (Toronto, Canada). Liquid chromatography grade of methanol and acetonitrile were purchased from Merck (Schwalbach, Germany). Ammonium acetate (99.99%) was obtained from Shanghai Macklin Biochemical Co. Ltd. (Shanghai, China). Formic acid (liquid chromatography grade, ≥98%), allyltriethoxysilane, vinylbenzenesulphonic acid (VA), glucose, dichloromethane, ethylene glycol dimethacrylate and 2,2′-azobis(2-methylpropionitrile) were obtained from J&K Chemical Ltd. (Beijing, China). Eleven types of scented tea, including Erdbeer-Sahne tea, Earl Grey tea, Magie des Orients tea, Himbeer-Vanille tea, Orange-Ingwer tea, Orange des Sudens tea, Klassisch tea, Mango-Kokos tea, Kamille tea, Jasmine tea and Sahne-Karamell tea were purchased from local supermarkets. Ultrapure water was generated by using a Milli-Q water purification system (Bedford, United States). Quartz fibers were supplied by Chuangli Optical Fiber (diameter: 125 μm, Shenzhen, China). Other chemicals were supplied by local supplier.

The UPLC-Q-TOF-MS system (Waters Corporation, United States) was used for the analysis. UPLC HSS T3 (1.0 × 100 mm, 1.8 μm) was used as the chromatographic column. Mobile phase A and mobile phase B were 0.1% formic acid in methanol and 0.2 mmol L^−1^ of ammonium acetate in water, respectively. A linear gradient program was used for the separation, and the sequence was set as: 0–3 min, 5% A; 3–8 min 10% A; 8–12 min, 50% A; 12–20 min, 70% A; 20–22 min, 95% A; 22–25 min, 5% A. The column temperature was set as 35°C. The flow rate was set as 0.3 ml min^−1^. The injection volume was 1 microliter of sample. The instrument was performed in positive electrospray mode (ESI^+^). Multiple-ion reaction monitoring mode was used for the analysis. Source temperature was set as 100°C. Cone gas flow rate was set as 50 L h^−1^. The desolvation and cone gas was high-purity N_2_. Desolvation temperature was set as 500°C. Desolvation gas flow rate was 800 L h^−1^. The key parameters, such as MRM transitions, cone voltage and capillary energy, are shown in Table 1.

**TABLE 1:** UPLC-Q-TOF-MS parameters of HPAs.

Compound	MRM ion transitions(*m/z*)		Cone voltage (V)	Capillary energy (kV)
	Confirmation ion	Quantitative ion		
lasiocarpine	412.02/336.20	412.20/120.05	28	1.8
monocrotaline	326.06/194.10	326.16/243.95	5	2.0
seneciphylline	335.10/120.08	334.16/156.15	19	2.0
retrorsine	352.18/120.08	352.18/311.20	9	2.0
echimidine	398.02/220.09	398.20/120.05	25	1.8
erucifoline	349.02/120.08	349.20/268.08	10	2.1
heliotrine	313.90/138.10	314.20/156.10	20	1.8
europine	329.90/120.10	330.20/138.05	23	1.8
lycopsamine	299.15/156.10	300.18/94.06	28	1.8
senecionine	336.20/138.20	336.20/282.20	15	2.0

### Preparation of multi-template imprinted molecularly imprinted solid phase microextraction fibers

**FIGURE 1:**
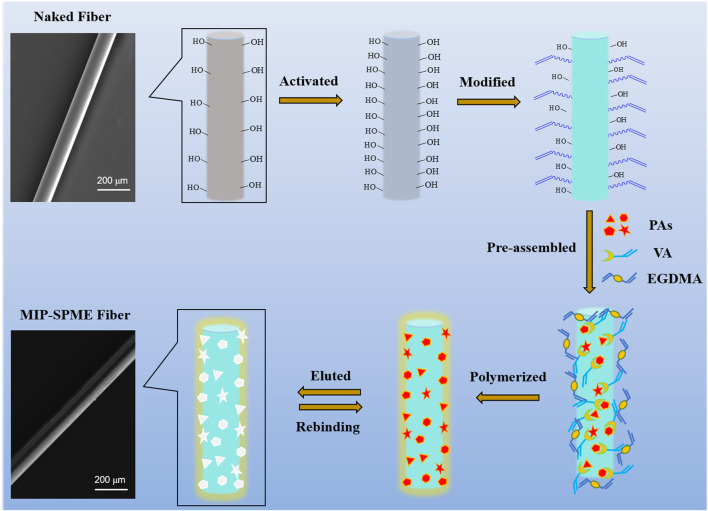
The schematic of preparation procedure of MIP-SPME fiber.

The schematic of MIP-SPME fiber preparation is shown in Figure 1. Briefly, naked quartz fibers were firstly immersed in concentrated sulfuric acid for activation, then they were washed with ultrapure water to neutral and dried at room temperature. The obtained fibers were modified for 6 h in a mixed solution which contained with 2 ml of allyltriethoxysilane, 1 ml of trimethylamine and 35 ml of toluene at 110°C. After siliconization, the modified fibers were washed with methanol and dried at room temperature. Next, the modified fibers were immersed in a mixed solution containing with 55 mg of VA, 8 mg of monocrotaline, 8 mg of senecionine, 8 mg of heliotrine, 8 mg of lycopsamine, 3 ml of methanol and 3 ml of acetonitrile for pre-polymerization. One hour later, the mixture was added with 0.5 mM of ethylene glycol dimethacrylate and 0.5 mM of 2,2′-azobis(2-methylpropionitrile) and mixed under 15 min of sonication. Then the nitrogen gas flow was blown in the mixed solution for the elimination of oxygen. After that, the whole reaction system was quickly sealed and the whole system was polymerized at 65°C for 12 h. Then obtained fibers were washed with the mixture of methanol and NH_3_⋅H_2_O (9:1, v/v) to remove the templates until no monocrotaline, senecionine, heliotrine and lycopasmine could be detected. Finally, the obtained MIP fibers were washed with methanol and water to remove the residual solvent and then dried at 40°C. The non-imprinted polymer (NIP) fibers were fabricated by the same process as MIP fibers but without the addition of the templates.

### Characterization of solid phase microextraction fibers

The morphologies of naked fiber, modified fiber and MIP fiber were recorded by a field emission scanning electron microscope (GeminiSEM 500, Carl Zeiss, Germany), and the chemical composition of each fiber was obtained simultaneously by the energy dispersive spectrometer and it was scanned in a square of 10 μm × 10 μm. The specific surface areas of modified fiber and MIP fiber were detected by using nitrogen adsorption and desorption analyses (Autochem2920, Auantachrome, United States) and were calculated according to the Brunauer-Emmett-Teller (BET) theory.

### Adsorption test

The obtained fibers were fixed and hanged in the solutions and 1 cm length of each fiber was soaked in the solution. They were respectively immersed in a single PA standard solution with the concentration range of 200–1,600 ng⋅mL^−1^ for the adsorption. After extraction, the fiber was taken off and rinsed with washing solution. After washing, the fiber was immersed in the eluting solvent with a vigorous stirring. After elution, the obtained eluting solvent was evaporated and reconstituted with methanol for UPLC-Q-TOF-MS analysis. The selectivity of MIP-SPME fiber was tested among ten HPAs (monocrotaline, erucifoline, lasiocarpine, retrorsine, europine, lycopsamine, heliotrine, seneciphylline, echimidine and senecionine), three non-alkaloids (naringin, luteolin and glucose) and two positive alkaloid control (sinomenine and berberine hydrochloride), and their chemical structures are shown in Supplementary Figure S1. The mixed solution that contained with these 15 types of compounds (400 ng⋅mL^−1^) was obtained by mixing each standard solution together to mimic real samples. The MIP and NIP fibers were respectively soaked in the mixed samples for adsorption. After certain minutes’ extraction, the fibers were taken off and then rinsed with washing solvent. Then the fibers were put into eluting solvent with continuous stirring. After desorbing, the fibers were removed and the obtained eluting solutions were evaporated and reconstituted with methanol for UPLC-Q-TOF-MS analysis. All of adsorptions were carried out by triplicate.

The adsorption capacity *Q* (ng) was calculated according to equation *Q* = (*C*_0_-*C*_f_) × *v*, where *C*_0_ (ng⋅mL^−1^) and *C*_f_ (ng⋅mL^−1^) are the initial and final concentration of target compounds in solution, respectively; *v* (ml) is the total volume of the solution. The imprinting effect of obtained polymers is evaluated by using imprinted factor (IF). *IF* was calculated according to equation *IF* = *Q*_MIP_/*Q*_NIP_, where *Q*_MIP_ and *Q*_NIP_ are the adsorption capacity of MIP fiber and NIP fiber, respectively. Technically, *IF* > 2 indicates a relatively good imprinting effect (Shi et al., 2012). Selectivity coefficient (*S*) was calculated according to equation *S* = *Q*_PAs_/*Q*_non-PAs_, where *Q*_PAs_ and *Q*_non-PAs_ are the adsorption amount of MIP fiber for PAs and other compounds, respectively.

### Extraction conditions

The key parameters were optimized to get the best extraction efficiency of MIP-SPME, including the extraction time, elution solvent and eluting time. Firstly, MIP-SPME fibers were washed with 0.5 ml of methanol and 0.5 ml of 0.05 mM sulfuric acid, respectively. Next, MIP-SPME fibers were soaked into 0.5 ml of test solutions and absorbed for 1, 5, 10, 15, 20, 30, and 60 min, respectively. After that, the fibers were taken off and rinsed with 0.5 ml of water. Then, 0.5 ml of methanol containing with 0–5% of ammonium hydroxide were used to elute the target compounds for 1, 5, 10, 15, 20, 30, and 60 min, respectively. Last, the obtained solutions were dried by nitrogen gas blow. The obtained residues were re-dissolved with 0.1 ml of methanol and centrifuged at 12000 rpm for 15 min. The obtained supernatants were analyzed by using UPLC-Q-TOF system.

## Method validation

According to the recommendations of the International Conference on Harmonization Q2(R1), the analytical method was validated. (Borman and Elder, 2017). Jasmine tea sample was detected without these ten types of HPAs, so the spiked Jasmine tea sample was used as the model sample. To evaluate the linearity of this method, the calibration curves were obtained in the linear range of 2–500 ng mL^−1^. The limit of detection (LOD) and limit of quantitation (LOQ) were estimated when the signal-to-noise (S/N) ratios were reached to 3:1 and 10:1, respectively. In order to evaluate the precision and recovery, six sets of spiked samples were tested at three different concentrations (5, 10, 50 ng mL^−1^). In order to evaluate the intra-day and inter-day repeatability, six sets of spiked samples were also estimated in a single day and for 3 days, respectively.

### Sample analysis

A high speed mixer was used for the pulverization of scented tea samples. The obtained powder was sieved by using 30 mesh of sifter. 5 g of each obtained scented tea sample were dispersed in 5 ml of 10% ammonium hydroxide. Five minutes later, 100 ml of methanol-dichloromethane (1:1) was poured in and 3 hours’ heating reflux was executed for the extraction. The obtained solutions were filtered and the residue was re-extracted by using the same mixed solution for three times. The extracted solutions were combined and evaporated by using rotary evaporation. The mixture of methanol and water (1:1) was used to re-dissolve the residues and the pH of obtained solution was adjusted with formic acid to neutral. The obtained solutions were kept at 4°C overnight and then centrifuged for 10 min at 6,000 rpm. The supernatants were separated and kept for MIP-SPME. After MIP-SPME treatment, the obtained samples were used for UPLC-Q-TOF-MS analysis.

## Results and discussion

### Preparation of multi-template molecularly imprinted solid phase microextraction fibers

Although MIPs have been widely used as the extraction media for sample pretreatment in recent decade, the real application of MIPs still encounters some challenges. One of challenges is the requirement of simultaneous detection of multicomponent targets in real sample analysis, especially from industrial point of view (Xie et al., 2019; Mohiuddin et al., 2021). However, much work mainly was devoted on the use of MIP for the extraction of one type of compound from complex matrix, which limited it from extracting and detecting a series of target molecules (Madikizela and Chimuka, 2016). HPAs were a series of compounds that were not only with the same necine parent nucleus structure but also simultaneously existed in plants. Therefore, four typical toxic HPAs were used as templates for the fabrication of multi-template MIP-SPME fibers in this work. It includes monocrotaline, senecionine, heliotrine and lycopasmine, in which the senecio acid part of heliotrine and lycopsamine are open-loop and that of moncrotaline and senecionine are closed-loop (as shown in Supplementary Figure S1). In this work, surface molecular imprinting technique was used for the fabrication of MIP fibers because it could provide large amount of accessible adsorption sites on the surface of MIP fibers. In the formation of MIPs, functional monomer plays a crucial role because it can directly affect the interaction and recognition ability of MIPs. Here, VA was used as the functional monomer, and the sulfonic anion group of VA could interact with the amine group and hydroxyl group of HPAs through strong ionic bond. Plus the effect of three-dimensional cavity matched with templates, these merits facilitate the specific recognition and vast adsorption of MIP fibers for target HPAs from complex matrix. It has been widely proven that MIPs were durable and robust matrix for a wide variety of applications and chemical environments (Pan et al., 2018; BelBruno, 2019). The supporter silica fibers also possess excellent thermal stability and minimal swelling property in the presence of solvents, facilitating the maintenance of the shape and size of imprint cavities (Lofgreen and Ozin, 2014). These merits will not only allow MIP fibers to specifically recognize the target compounds but also facilitate the wide usage in the complex matrix.

### Characterization of different fibers

**FIGURE 2:**
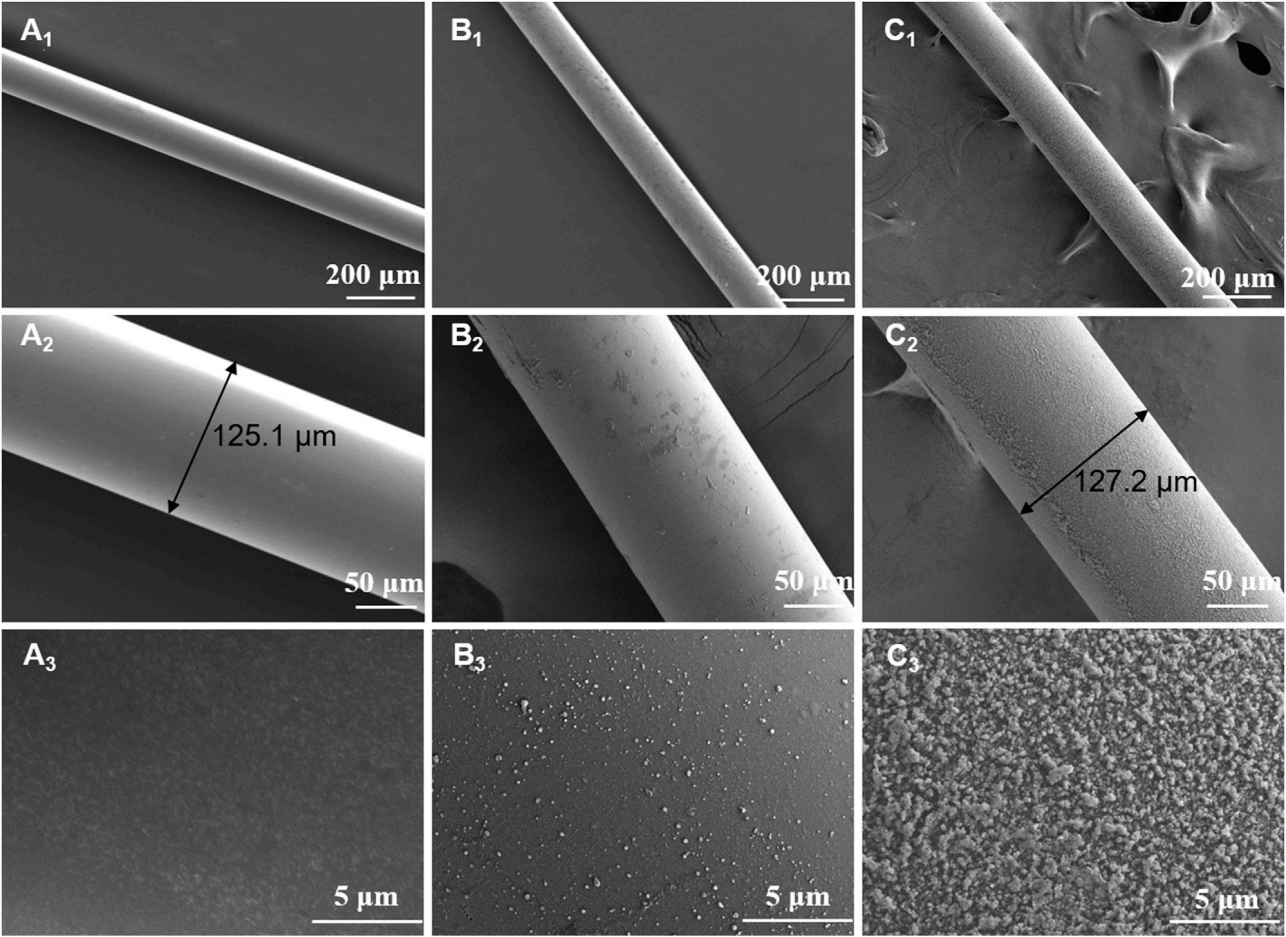
SEM images of naked fiber **(A**_**1**_ **∼A**_**3**_ **)**, modified fiber **(B**_**1**_ **∼B**_**3**_ **)** and MIP fiber **(C**_**1**_ **∼C**_**3**_ **)** with different magnifications.

As shown in Figure 2, the surface morphology of naked fiber, modified fiber and MIP fiber were quite different. The surface of naked fiber was much smooth and without any attachments. After modification, the surface of modified fiber became much rougher than that of naked fiber, indicating the successful silanization. After polymerization, a homogeneous imprinted layer was successfully grafted on the surface of modified fiber. The diameter of naked fiber and MIP fiber was about 125 μm and 127 μm respectively, indicating that the thickness of grafted MIP layer was about 1 μm. The thickness of MIP layer would affect the extraction efficiency of MIP fibers, therefore, the key parameters should be fixed during polymerization, such as the polymerization time, the concentrations of functional monomer and crosslinker, etc. When magnifying the surface of MIP fiber, it was also bumpy, which was highly benefit to increase the surface area and improve the adsorption capacity. The specific surface areas of modified fiber and MIP fiber were also detected and the values of them were 67.15 m^2^ g^−1^ and 289.43 m^2^ g^−1^ respectively. The results indicated that the specific surface area of fibers were greatly improved after grafted with MIP layer, which would be highly beneficial to the increase of adsorption capacity of MIP fibers. The element composition that existed on the surface of different fibers was also distinct from each other. As shown in Table 2, the percentage of carbon on the surface of modified fiber was increased after grafting allyltriethoxysilane compared with naked fiber, indicating the successful modification. The presence of sulfur element on the surface of MIP fiber further indicated the successful imprinting.

**TABLE 2:** Elementary analysis results of different samples.

Sample\element	C	N	O	S	Si
Naked fiber	6.24	3.49	56.11	ND	34.16
Modified fiber	22.36	4.52	44.15	ND	28.97
MIP fiber	39.48	1.77	42.22	4.39	12.14

ND: not detected.

### Adsorption test

**FIGURE 3:**
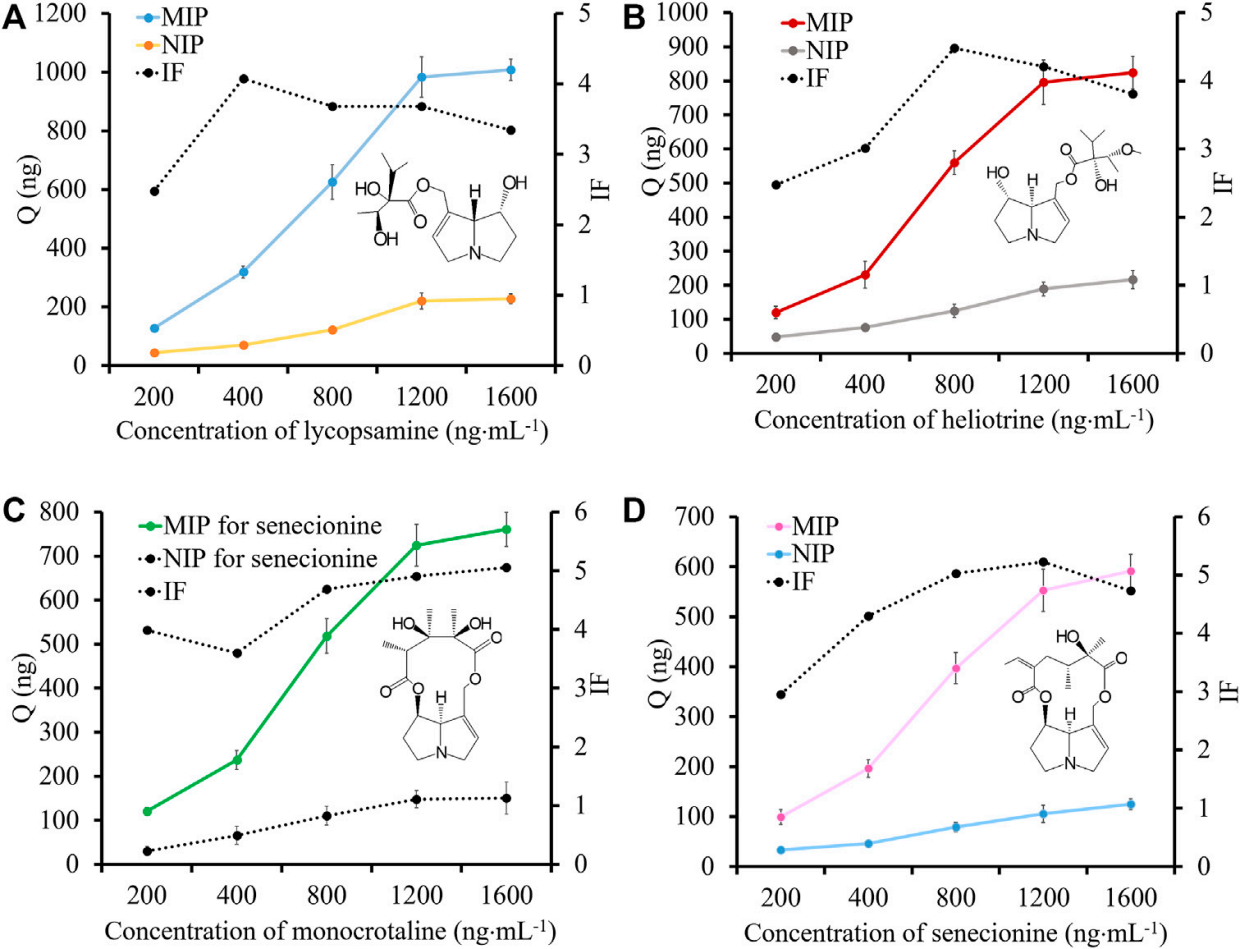
The adsorption capacity and IF values of MIP fibers and NIP fibers for lycopsamine **(A)**, heliotrine **(B)**, monocrotaline **(C)** and senecionine **(D)**.

The adsorption capacity of MIP for four templates were studied. The results are shown in Figure 3. At the concentration of 1,600 ng⋅mL^−1^, the adsorption capacity of MIP fiber for different templates was varied from 591.1 ng to 1,007.8 ng. Generally, the adsorption ability of MIP fiber for lycopsamine and heliotrine was higher than that for monocrotaline and senecionine. The possible reason was the difference of the senecio acid part of them. The open-loop senecio acid part of lycopsamine or heliotrine endows hydroxyl group and necine group with much flexibility, which facilitate their accessibility to interact with the sulfonic acid group of MIP. However, the IF values of monocrotaline and senecionine were slightly higher than that of lycopsamine and heliotrine. This might because the rigid structure could promote the imprinting effect. In summary, the IF values were ranged from 2.15 to 5.22, indicating that MIP fiber showed high recognition ability for four templates.

**FIGURE 4:**
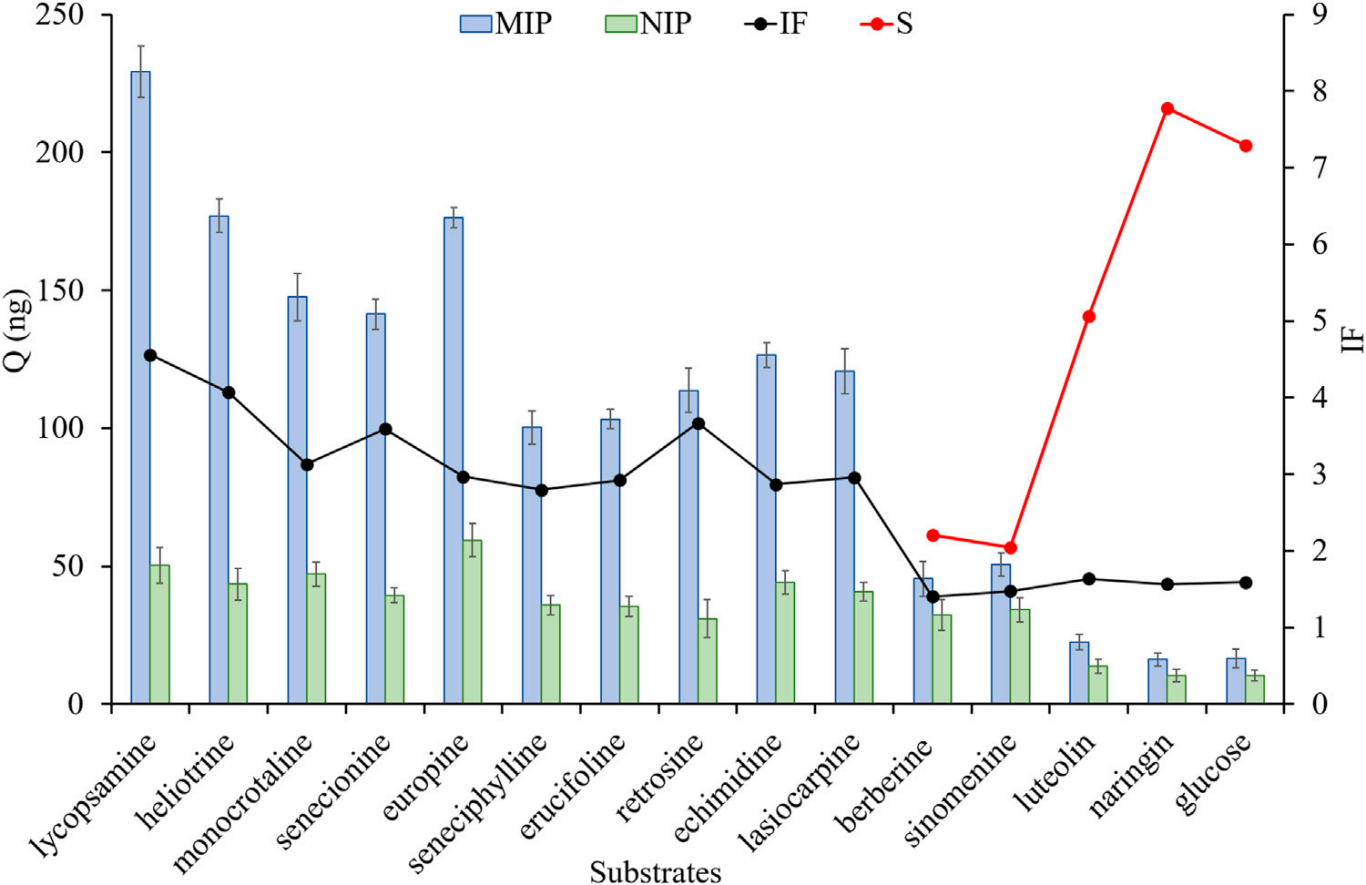
The adsorption capacity, imprinted factor and selectivity coefficient of different fibers for different substrates. The adsorbed solution was a mixture and was contained with 15 types of compounds, including lycopasmine, heliotrine, monocrotaline, senecionine, europine, seneciphylline, erucifoline, retrosine, echimidine, lasiocarpine, berberine, sinomenine, luteolin, naringin and glucose. The concentration of each compound was 400 ng⋅mL^−1^.

In order to further extend the usability and verify the selectivity of MIP fibers, the mixed solution was used as the test sample, which contained with ten HPAs, two other kind of alkaloid compounds, two flavonoid compounds and glucose (As shown in Figure 4). The results showed that the adsorption abilities of MIP fibers for ten HPAs were all over 100 ng, in which the adsorption capacity of MIP for seneciphylline was the lowest (100.3 ng). Nevertheless, it was still much higher than that for non-HPA compounds (ranged from 10.4 to 34.2 ng). Moreover, the IF values of ten HPAs were all over 2.8 and IF values of five non-HPAs were all lower than 1.6, demonstrating that MIP fiber exhibited high recognition ability and selectivity for these ten HPAs simultaneously. The selectivity coefficient (*S*) was calculated by using *Q*_seneciphylline_/*Q*_non-HPAs_, and *S* values were ranged from 2.1 to 7.8, suggesting that MIP fiber showed excellent specific selectivity for HPAs. It might be ascribed to the specific recognition ability of MIPs for the analogues of templates. Therefore, the fabricated MIP fibers would be used for the specific recognition and selective adsorption of these ten HPAs.

Our previous research has reported a single template MIP fiber for selective adsorption of 4 PAs in herbal medicines (Luo, et al., 2019). In order to confirm the advantages of multi-template MIP fibers, the comparisons of IF and adsorption capacity between single template MIP and multi-template MIP are summarized in Table 3. IFs of most HPAs were improved more or less when the multi-template MIP fiber was used. The possible reason was that the cavities imprinted by four types of templates could provide much more feasible and accessible sites than that imprinted by single template. In addition, the increased template amount was also benefit for the increase of accessible sites and the IF values. Moreover, the adsorption capacities of multi-template MIP for these ten HPAs were greatly increased compared with that of single template MIP. There were two possible reasons: ① 200 μM of allylsulfonate and 50 μM of monocrotaline were used for the fabrication of single template MIP fiber, while 300 μM of VA and total 100 μM of template molecules were used for the fabrication of multi-template MIP fiber. The increased amounts of functional monomer and template molecules might generate much more accessible cavities. ②Compared with allylsulfonate based MIP fiber, vinylbenzenesulphonic acid based MIP fiber might be not only based upon the electrostatic interaction but also based on steric recognition or π-π conjugate interactions for the recognition of targets (Cudjoe and Pawliszyn, 2014).

**TABLE 3:** Comparison of IF and adsorption capacity between single template MIP fibers and multi-template MIP fibers.

Analytes	IF		Adsorption capacity (ng)	
	Single template MIP	Multi-template MIP	Single template MIP	Multi-template MIP
echimidine	2.43	2.87	40.05	126.61
erucifoline	1.98	2.93	14.81	103.30
europine	2.58	2.97	45.82	176.39
heliotrine	4.70	4.07	41.65	177.05
lycopsamine	1.05	4.56	12.53	229.25
lasiocarpine	4.54	2.96	56.71	120.62
retrosine	1.57	3.67	13.46	113.77
senecionine	1.90	3.59	16.11	141.38
seneciphylline	1.81	2.79	20.89	100.27
monocrotaline	4.92	3.13	89.54	147.62

### Optimization of solid phase microextraction procedure

**FIGURE 5:**
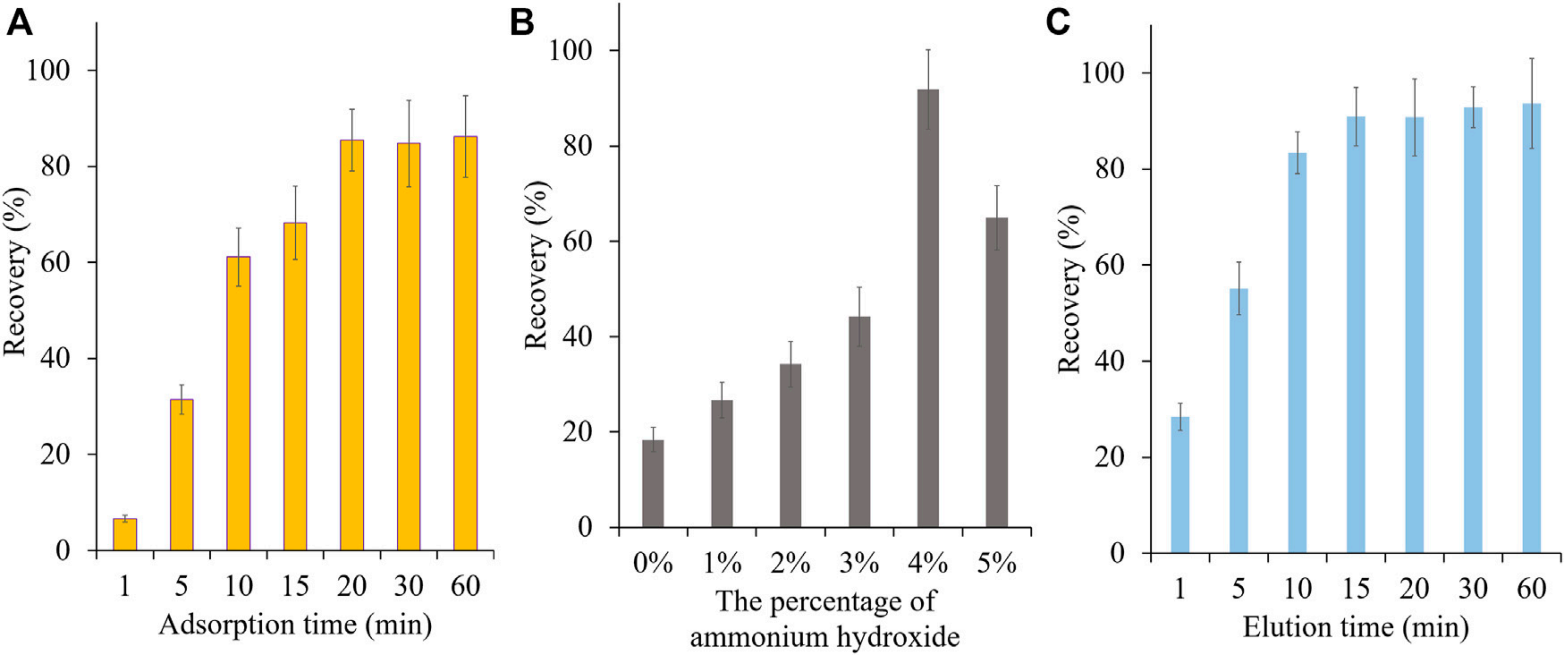
Optimization of MIP-SPME conditions. **(A)** Effect of adsorption time; **(B)** Effect of elution solvent; **(C)** Effect of elution time.

**FIGURE 6:**
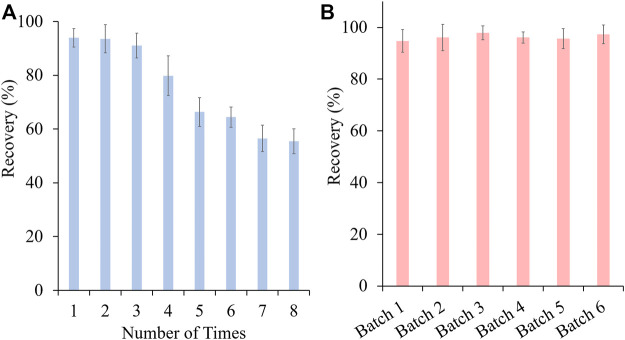
The recovery of different reused times **(A)** and different batches **(B)** of MIP fibers.

In this study, vinylbenzenesulphonic acid was used as the functional monomer and the sulfonic acid group of it would specifically interact with PAs. Before use, SPME fibers were preconditioned with methanol and dilute sulfuric acid respectively to remove the impurities and to activate the cation group. To ensure high extraction efficiency of MIP-SPME fibers, some crucial factors were optimized, including the adsorption time, eluting solvent and elution time. In order to reach the saturated adsorption, MIP-SPME fibers were immersed into the test samples for 1, 5, 10, 15, 20, 30, and 60 min adsorption at room temperature, respectively. As shown in Figure 5A, the recovery of total HPAs was highly increased with the increase of adsorption time. When the adsorption time was reached to 20 min, the adsorption equilibrium was obtained. Considering the stirring speed would affect the diffusion of compounds, the stirring speeds were also studied during adsorption test. In view of the fragile property of quartz fiber, a vigorous stirring was avoided. The stirring speed was set as 80, 100 and 120 rpm, respectively. The results showed less changes of adsorption capacity of MIP fibers. Therefore, 100 rpm was used in the whole experiment. The elution solvent consisted of acetonitrile and ammonium hydroxide. The eluting efficiency was shapely affected by the percentage of ammonium hydroxide in elution solvent. The possible reason was that the ionic bond between sulfonic anion group of MIP fibers and tertiary amine/hydroxyl group of HPAs was the main interaction when the adsorption happened. All of tested HPAs were with the same alkaline parent nucleus structure—necine, and the difference of p*K*a value of these HPAs were less than 0.59. Therefore, the elution of these HPAs were with the similar manner. In Figure 5B, with the increase of ammonium hydroxide, HPAs would be gradually changed as free state, and the ionic bonds would be broken. The free state of HPAs was then dissolved into acetonitrile step by step. When the percentage of ammonium hydroxide was increased to 5%, the recovery of total HPAs was decreased sharply. PAs were prone to be degraded when the solution was with a high concentration of ammonium hydroxide (Chung and Lam, 2018). Therefore, this phenomenon might because peralkalic solution would break the closed-loop senecio acid part of HPAs, which directly resulted in the decrease of tested HPAs. Figure 5C shows the effect of desorption time on the test, the recovery of total HPAs was increased shapely with the increase of elution time. After 15 min desorption, the recovery of total HPAs could be reached to the equilibrium. Even further prolong the elution time to 60 min, the recovery of total HPAs has no significant improvement. Therefore, 20 min of the adsorption time, acetonitrile with 4% ammonium hydroxide as elution solvent and 15 min of the elution time were optimized as the best MIP-SPME conditions. In order to investigate the reuse potential, MIP fibers were reused for 8 times and the results are shown in Figure 6A. The recovery of total HPAs was gradually decreased with the increase of cycle time. After 3 times usage, the recovery could be kept in 91.1%, indicating the relatively good reusability. The precision of six batches of MIP fibers was also studied and the results are shown in Figure 6B. The results showed that the precision of different batches of MIP fibers was relatively good (RSD = 1.2%).

### Method validation

**FIGURE 7:**
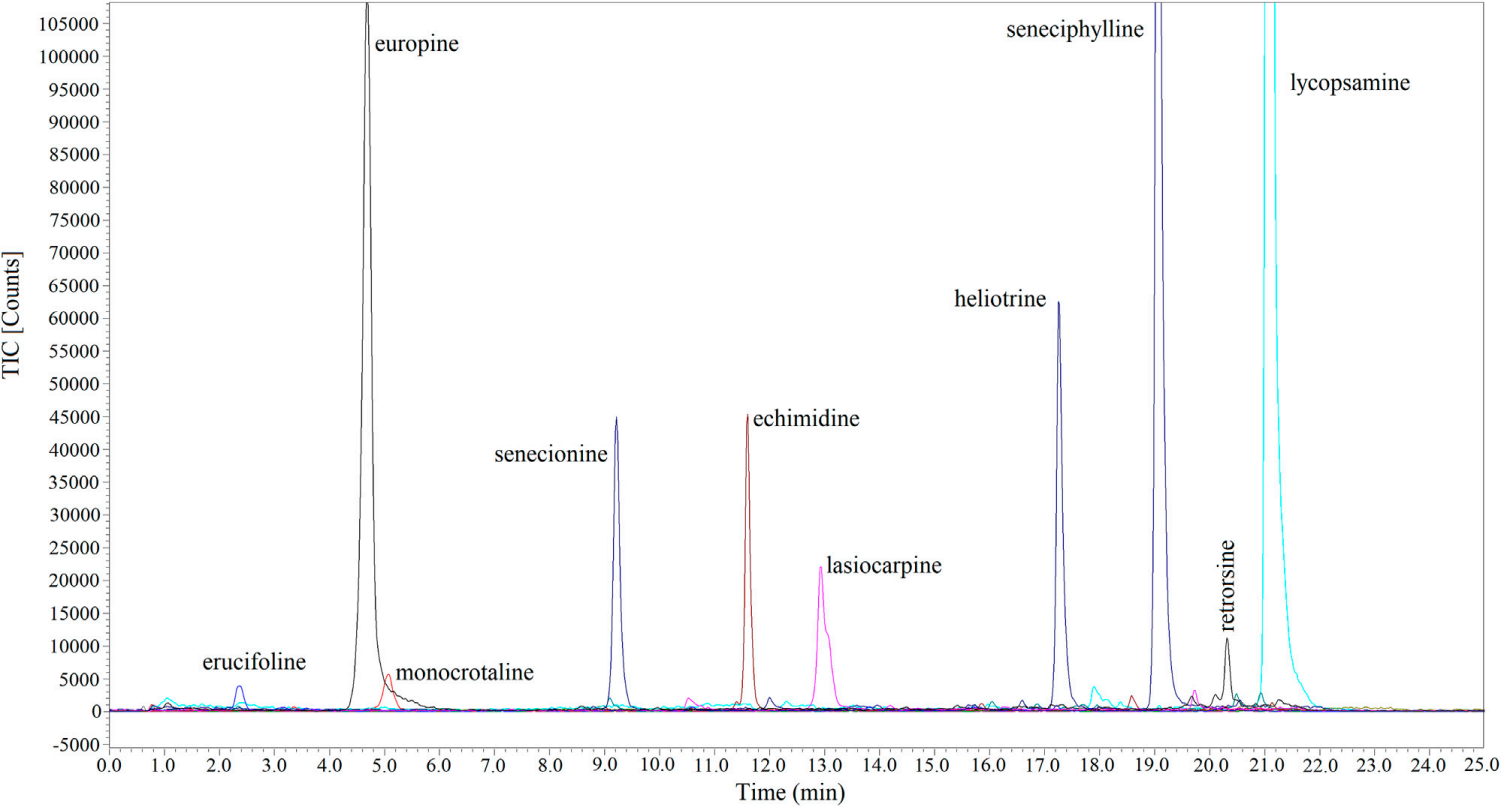
Topical ion chromatograms of ten HPAs in spiked Jasmine tea samples after MIP-SPME treatment.

Combined with UPLC-Q-TOF-MS, a method for the selective enrichment and sensitive detection of ten HPAs in scented tea samples was established by using MIP fiber as SPME material. The specificity, linearity, LOD, LOQ, accuracy, precision and reproducibility were investigated for the method validation. The specificity of the method was examined by using HPA spiked Jasmine tea sample (10 ng mL^−1^ of each compound). The topical ion chromatogram of these ten HPAs is shown in Figure 7, and the results showed that the spiked sample was clean enough after the treatment of MIP-SPME. The regression equations, the linear regression coefficients (*r* ^2^), LODs and LOQs of these compounds were summarized in Table 4. The results showed that the favorable linear relationships were obtained (*r* ^2^ values > 0.999) in a wide range of 2–500 ng mL^−1^. LODs of these HPAs were in the range of 0.08–0.54 ng mL^−1^ and LOQs of these HPAs were in the range of 0.26–1.77 ng mL^−1^. The results demonstrated that the established method was with high sensitivity for the determination of each HPA. Table 5 shows the results of accuracy and precision. It can be seen that the recovery of each compound at three levels was in the range of 90.7%–107.6% and relative standard deviation (RSD) was less than 10.4%. The results implied that this method was with satisfactory accuracy, precision and reproducibility. All of these results demonstrated that the established method was reliable and could be used for the sensitive and accurate determination of ten HPAs in scented tea samples.

**TABLE 4:** Analytical parameters of the proposed method.

Compound	Regression equation	*r* ^2^	LOD (ng·mL^−1^)	LOQ (ng·mL^−1^)
lasiocarpine	y = 1,650.7x + 3,392.1	0.9993	0.25	0.84
echimidine	y = 6,623.5x + 2,540.0	0.9995	0.24	0.96
senecionine	y = 2,548.2x + 5,539.7	0.9997	0.22	0.78
heliotrine	y = 6,234.1x + 3,057.5	0.9997	0.11	0.37
seneciphylline	y = 1,432.5x + 7,560.29	0.9996	0.10	0.32
retrorsine	y = 599.44x + 482.43	0.9999	0.27	1.03
lycopsamine	y = 5,099.1x + 42178	0.9995	0.08	0.26
europine	y = 2,750.9x + 4,790.6	0.9999	0.09	0.30
erucifoline	y = 613.09x + 114.4	0.9991	0.54	1.77
monocrotaline	y = 157.46x + 5,807.4	0.9990	0.47	1.58

**TABLE 5:** Accuracy and precision results summary for spiked scented tea samples.

Analytes	Accuracy (mean% *n* = 6)					Repeatability (RSD% *n* = 6)		Reproducibility (RSD% *n* = 6)	
	5 (ng·mL^−1^)	10 (ng·mL^−1^)	50 (ng·mL^−1^)	5 (ng·mL^−1^)	10 (ng·mL^−1^)	50 (ng·mL^−1^)	5 (ng·mL^−1^)	10 (ng·mL^−1^)	50 (ng·mL^−1^)
lycopsamine	96.6	94.6	93.8	6.6	4.6	3.8	7.2	2.5	3.3
europine	94.4	98.4	99.0	4.4	3.4	10.0	2.9	3.4	5.4
retrosine	92.0	98.5	99.6	7.9	2.5	9.6	3.9	4.1	4.4
seneciphylline	95.2	96.5	95.2	2.2	6.6	5.1	1.2	1.3	2.6
echimidine	96.8	99.7	93.6	6.7	9.7	3.5	7.5	5.7	6.7
lasiocarpine	92.4	90.7	107.6	2.4	5.7	7.6	2.0	3.2	4.3
senecionine	95.8	91.9	93.1	5.8	1.8	3.0	2.0	3.6	4.7
erucifoline	94.0	98.7	103.7	3.9	8.6	3.6	4.9	3.5	5.8
heliotrine	92.0	93.5	100.2	3.9	3.4	3.2	10.4	9.6	3.1
monocrotaline	98.1	93.9	99.3	5.4	6.7	3.3	9.8	8.3	3.5

### Detection of HPAs in scented tea samples

In recent 10 years, more and more studies reported that PAs were detected in different kinds of tea samples (Bodi, et al., 2014; Schulz, et al., 2015; Chen, et al., 2019; Casado, et al., 2022). Therefore, the safety of tea samples must deserves high attention. As shown in Supplementary Figure S2, scented tea samples are usually a mixture of tea, flower, fruit and spice, which are composed of large amount of different compositions. Additionally, a lot of sugar are added in the scented tea to cater the public taste. Therefore, how to reduce the matrix effect and simultaneously purify the trace amount of HPAs become the first important issue during the analysis procedure. There are four types of HPAs detected in Magie des Orients tea sample, including europine, lycopsamine, echimidine and heliotrine. Supplementary Figure S3 shows the comparison of total ion chromatograms of HPAs in Magie des Orients tea samples before and after MIP-SPME treatment. Compared with Supplementary Figures S3A1∼A4, the interference of matrices showed in Supplementary Figures S3B1∼B4 was efficiently removed and the target HPAs was well separated and enriched after MIP-SPME treatment. Consequently, MIP-SPME showed high selective extraction and excellent enrichment efficiency for the detection of HPAs in complex samples. Table 6 shows the comparison of different methods for the detection of PAs in different kinds of samples. The results showed that the LODs of these methods without of clean-up steps were higher than that of methods with SPE or SPME clean-up steps. It indicated that SPE or SPME treatment could highly improve the sensitivity of the detection of PAs in complex matrix. Compared with conventional SPE, the treatment time and the organic solvent consumption of MIP-SPME were all less than those of SPE, indicating the superior properties of MIP-SPME fibers.

**TABLE 6:** Comparison of different methods for detection of PAs in different samples.

Samples	Analytes	Methods	Time for clean-up (min)	Organic solvent used in clean-up (ml)	LOD (ng⋅mL^−1^)	Recovery (%)	References
*Senecio* and *Tussilago* genera	Senecionine, seneciphylline, retrorsine, senkirkine	Micellar electrokinetic chromatography	NM	NM	1,200–2,700	NM	Yu et al. (2005)
*Senecio scandens*	Adonifoline, senecionine, seneciphylline, lycopsamine, echimindine	LC-MS/MS	NM	NM	0.115	83.2–114.5	Wang D et al. (2020)
*Senecio scandens*	adonifoline	LC-MS/MS	NM	NM	0.5	96.9–97.7	Zhang et al. (2008)
*Crotalaria spectabilis*	monocrotaline	LC-MS/MS	NM	NM	70	108–114	Scupinari et al. (2020)
Teas and herbal teas	lycopsamine, retrorsine	SPE and LC-MS/MS	> 60	> 7	0.5	NM	Mathon et al. (2014)
sorghum, oregano, herbal teas	echimidine, echinatine, erucifoline, europine, heliotrine, indicine, intermedine, jacobine, lasiocarpine, lycopsamine, monocrotaline, retronecine, retrorsine, senecionine, seneciphylline, senecivernine, senkirkine, and trichodesmine	Dispersive solid-phase extraction and LC-MS	> 60	> 2	0.5–10	78–117	Dzuman et al. (2020)
Honey	atropine, scopolamine, echimidine, heliotrine, intermedine, lasiocarpine, lycopsamine, retrorsine, senecionine, seneciphylline, senkirkine	QuEChERS method and LC-MS	26	10	0.04–0.2	92.3–114.8	Martinello et al. (2017)
Lipid foodstuffs	crotalinein, intermedine, retrorsine, heliotrine, senecipylline, senecionine, echimidine, senkirkine, lasiocarpine	SCX-SPE and LC-MS/MS	40	10.5	0.06–0.6	81.6–104.9	Yoon et al. (2015)
*Gynura japonica*	monocrotaline, senecionine, seneciphylline, retrorsine, adonifoline, senkirkine	Liquid-liquid extraction and real-time MS	NM	>10	0.55–0.85	89.3–112.1	Chen et al. (2021)
Scented teas	senecionine, lycopsamine, retrorsine, heliotrine, lasiocarpine, monocrotaline, echimidine, erucifoline, europine and seneciphylline	MIP-SPME and LC-MS	35	1	0.08–0.54	90.7–107.6	This work

NM: not mentioned.

**FIGURE 8:**
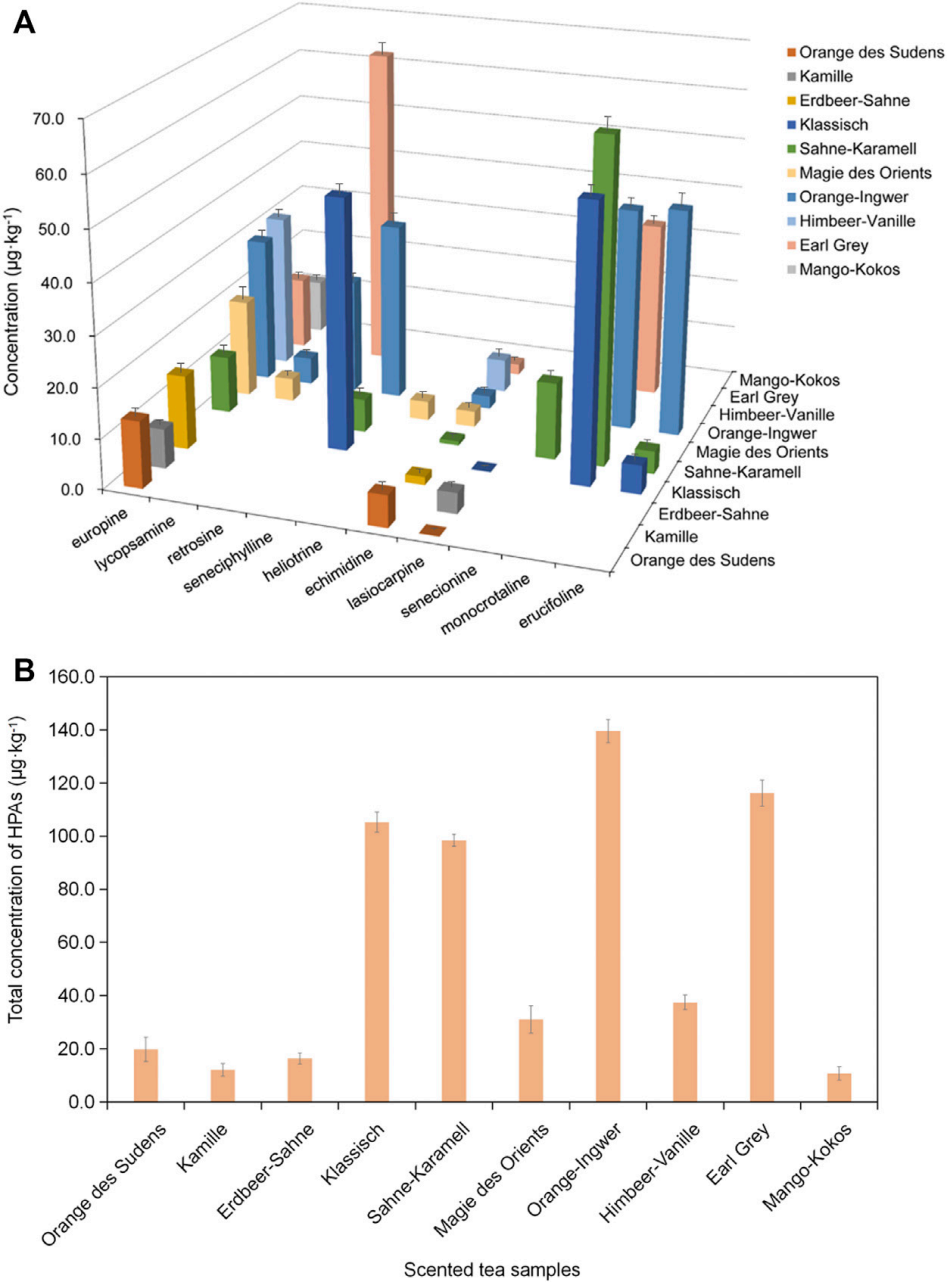
The concentration of HPAs in scented tea samples. **(A)** the concentration of a single analyte in positive tested samples; **(B)** the total concentration of HPAs in positive tested samples.

In this work, eleven types of scented tea samples were analyzed by using established UPLC-Q-TOF-MS coupled with MIP-SPME method, and ten of them were positive and only Jasmine tea sample was without of these ten HPAs. As shown in Figure 8A, the concentration of a single analyte in positive tested samples was ranged from 0.1 to 64.5 μg kg^−1^, and europine and echimidine were frequently existed in these tested samples. Lorena *et al* reported that echimidine was also most frequently existed in a series of honey samples (Lorena et al., 2016). Figure 8B shows that Orange des Sudens tea, Erdbeer-Sahne tea, Magie des Orients tea, Himbeer Vanille tea, Mango-Kokos tea and Kamille tea were tested with relatively lower concentration of HPAs (19.9, 16.4, 31.0, 37.5, 10.8, and 12.1 μg kg^−1^, respectively). The most diverse and highest total concentration of HPAs (139.6 μg kg^−1^) was detected in Orange-Ingwer tea samples. The total concentrations of HPAs in Earl Grey tea, Sahne-Karamell tea and Klassisch tea were also much higher and were 116.3, 98.5 and 105.3 μg kg^−1^, respectively. Usually, one package of scented tea was about 2 g. According to the guidance value in EFSA (EFSA, 2011), a daily intake of PAs should be no more than 0.007 μg kg^−1^ per day, which means that the daily intake of Orange-Ingwer tea, Earl Grey tea, Sahne-Karamell tea and Klassisch tea should be less than two packages, or else, they will present a potential risk to human health.

## Conclusion

In this study, MIP-SPME fibers were prepared by using multi-template imprinting technique for selective extraction and specific enrichment of HPAs in scented tea samples. MIP-SPME fibers exhibited excellent specific recognition ability and selectivity for ten types of HPAs. Then MIP-SPME coupled with UPLC-Q-TOF-MS method was established for sensitive and accurate determination of ten HPAs in scented tea samples. The established method exhibited superior advantages, including accuracy, simplicity and selectivity, which makes the reliable determination and confirmation of ten types of HPA in scented tea samples possible. The established method was used for systematically studying the types and the concentrations of HPAs in ten types of popular scented tea samples. Due to the variety compositions in scented tea, different scented tea samples contained with different types and concentrations of HPAs. Considering the safety threats of HPAs, the implementation of much more rigorous quality control tests is necessary to minimize possible ingestion of HPA-contaminated scented tea.

## Data Availability

The original contributions presented in the study are included in the article/Supplementary Material, further inquiries can be directed to the corresponding authors.

## Supplementary material

Supplementary Figure S1 Chemical structure of ten HPAs and five other compounds.

Supplementary Figure S2 The images of different types of scented tea. **(A)**: Erdbeer-Sahne; **(B)**: Earl Grey; **(C)**: Magie des Orients; **(D)**: Himbeer Vanille; **(E)**: Orange-Ingwer; **(F)**: Orange des Sudens; **(G)**: Klassisch; **(H)**: Mango-Kokos; **(I)**: Kamille; **(J)**: Sahne-Karamell.

Supplementary Figure S3 Total ion chromatograms of HPAs in Magie des Orients tea samples before **(A1∼A4)** and after **(B1∼B4)** MIP-SPME treatment.
