# Supplementary figures and images for: Multi-template imprinted solid-phase microextraction coupled with UPLC-Q-TOF-MS for simultaneous monitoring of ten hepatotoxic pyrrolizidine alkaloids in scented tea
Source: Front Chem. 2022 Nov 28;10:1048467. doi: 10.3389/fchem.2022.1048467 (PMC9742424; doi:10.3389/fchem.2022.1048467)

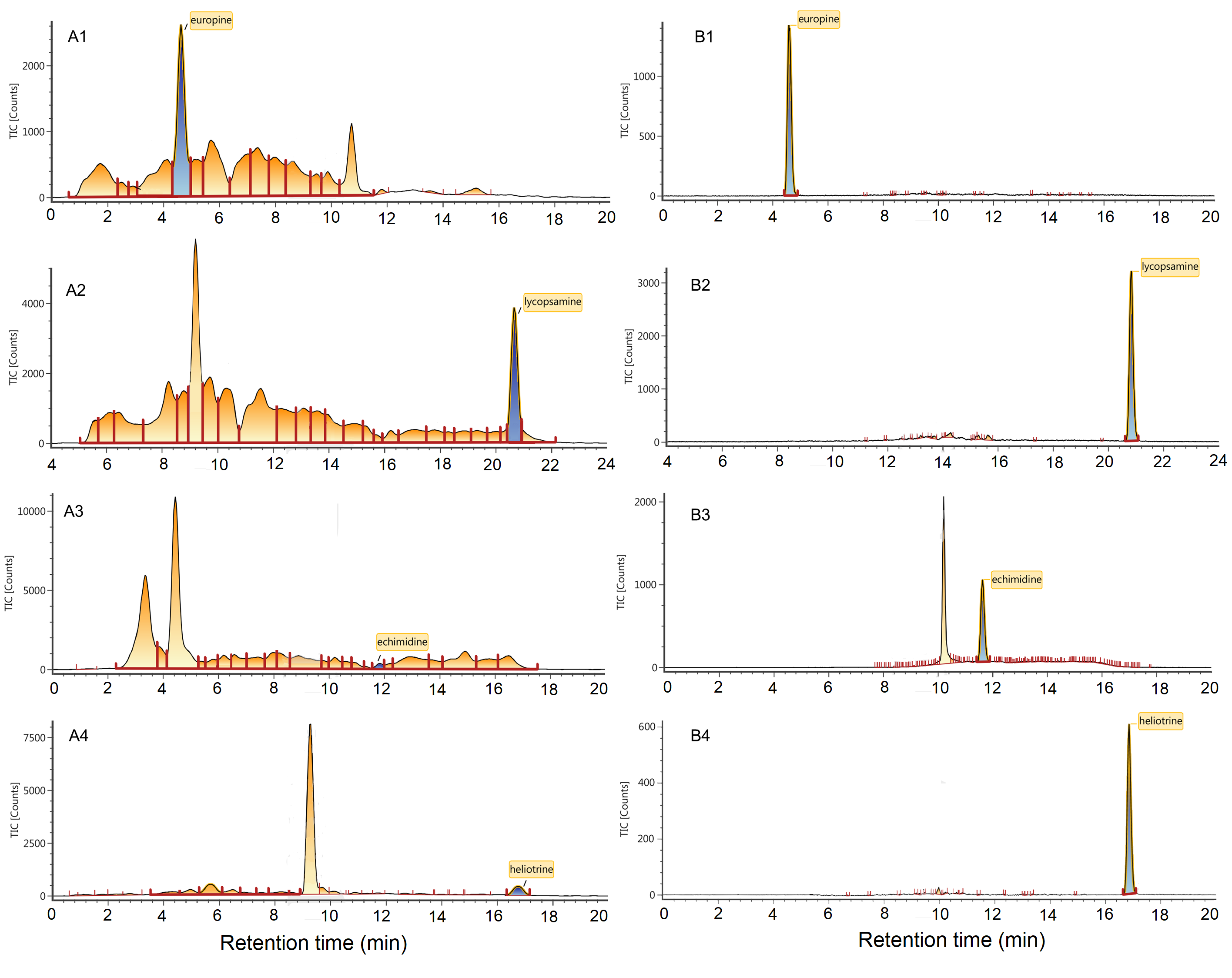

Supplement: Supplementary file 1 [file Image3.TIF]

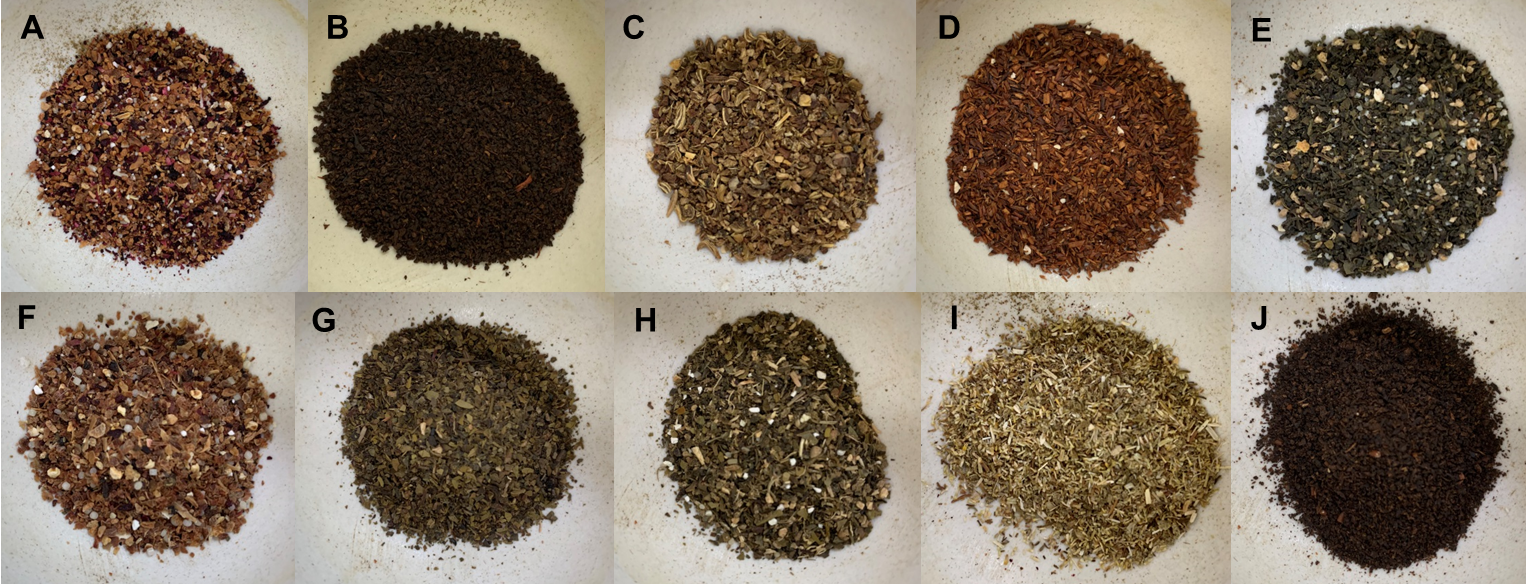

Supplement: Supplementary file 2 [file Image2.TIF]

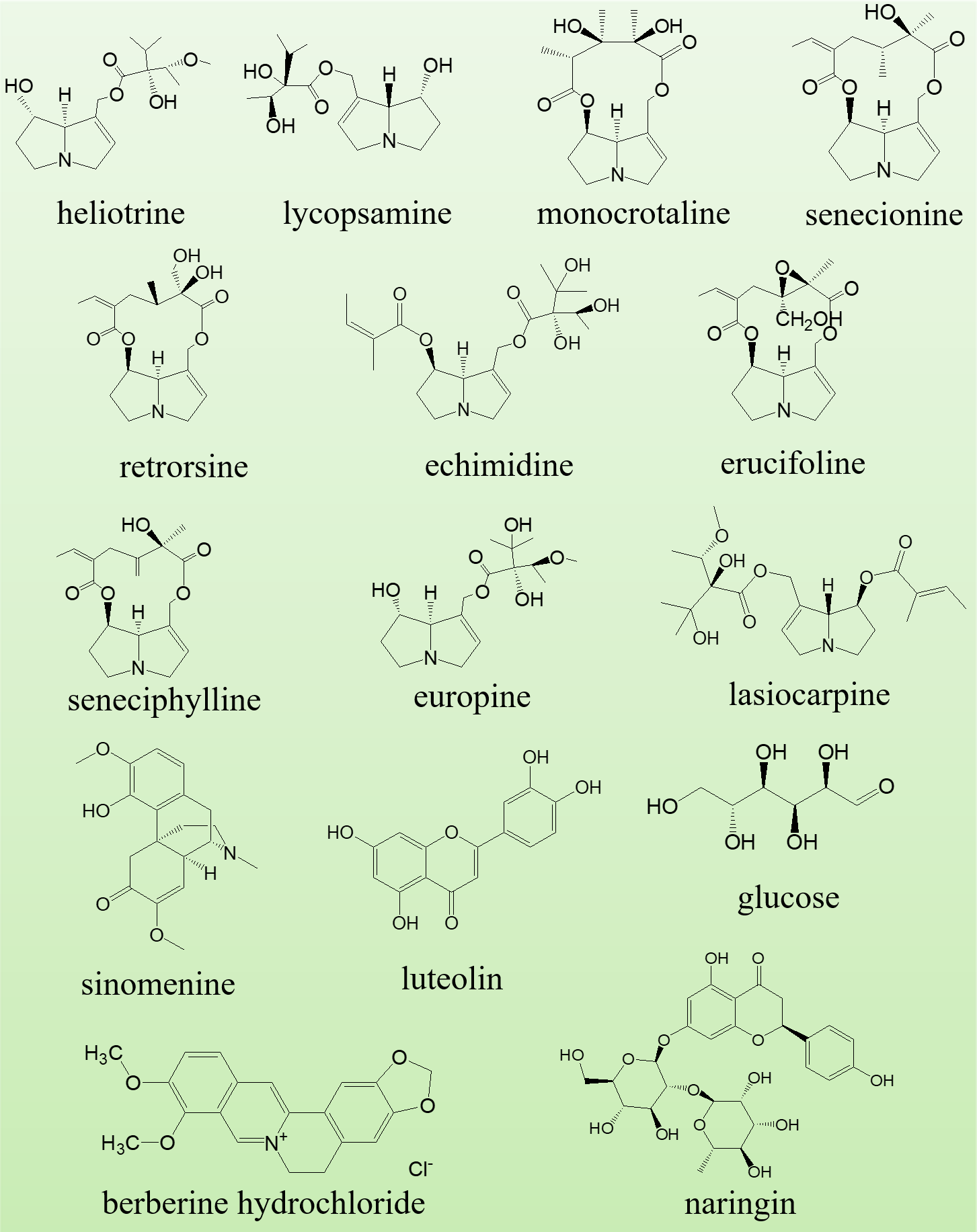

Supplement: Supplementary file 3 [file Image1.TIF]
